# One-Pot Reverse Transcriptional Loop-Mediated Isothermal Amplification (RT-LAMP) for Detecting MERS-CoV

**DOI:** 10.3389/fmicb.2016.02166

**Published:** 2017-01-09

**Authors:** Se Hee Lee, Yun Hee Baek, Yang-Hoon Kim, Young-Ki Choi, Min-Suk Song, Ji-Young Ahn

**Affiliations:** ^1^School of Biological Sciences, Chungbuk National UniversityCheongju, South Korea; ^2^College of Medicine and Medical Research Institute, Chungbuk National UniversityCheongju, South Korea

**Keywords:** one-pot reverse transcription (RT), MERS-CoV, loop-mediated isothermal amplification (LAMP), nucleocapsid (N) protein, EvaGreen

## Abstract

Due to the limitation of rapid development of specific antiviral drug or vaccine for novel emerging viruses, an accurate and rapid diagnosis is a key to manage the virus spread. We developed an efficient and rapid method with high specificity for the Middle East Respiratory Syndrome coronavirus (MERS-CoV), based on one-pot reverse transcription loop-mediated isothermal amplification (one-pot RT-LAMP). A set of six LAMP primers [F3, B3, FIP, BIP, LF (Loop-F), and LB (Loop-B)] were designed using the sequence of nucleocapsid (N) gene with optimized RT-LAMP enzyme conditions: 100 U M-MLV RTase and 4 U *Bst* polymerase, implying that the reaction was able to detect four infectious viral genome copies of MERS-CoV within a 60 min reaction time period. Significantly, EvaGreen dye has better signal read-out properties in one-pot RT-LAMP reaction and is more compatible with DNA polymerase than SYBR green I. Isothermally amplified specific N genes were further evaluated using field-deployable microchamber devices, leading to the specific identification of as few as 0.4 infectious viral genome copies, with no cross-reaction to the other acute respiratory disease viruses, including influenza type A (H1N1 and H3N2), type B, human coronavirus 229E, and human metapneumovirus. This sensitive, specific and feasible method provides a large-scale technical support in emergencies, and is also applied as a sample-to-detection module in Point of Care Testing devices.

## Introduction

A novel coronavirus, later defined as Middle East Respiratory Syndrome coronavirus (MERS-CoV), was first reported from an isolate of a patient who had died of severe pneumonia in Saudi Arabia in September 2012 (Zaki et al., 2012). As of August 31st 2016, the virus has infected 1,800 humans, with about 35% mortality rate, in 27 countries^1^. The newly emerging virus has been majorly causing human infections in countries in the Middle East, and cases outside the area are related to travelers to Arabian Peninsula or their contacts (Mailles et al., 2013; Bialek et al., 2014; Bin et al., 2016). Despite the limited human to human transmission, the high mortality rate and a recent large nosocomial outbreak in South Korea in 2015 confers a burden that the virus could efficiently transmit in the social community with severe disease outcomes. Thus, the development of effective therapeutics and rapid diagnostic tools are urgently needed to control the spread of MERS-CoV. However, due to the limitations to rapid development of vaccines and specific antiviral drugs for the newly emerging virus, diagnosis is the key to prevent or delay the viral spread.

Middle East Respiratory Syndrome coronavirus is a single stranded RNA virus, approximately 30 kb and is closely related to the bat coronaviruses in group 2c betacoronavirus, the severe acute respiratory syndrome coronavirus (SARS-CoV), reported to have caused 8,098 infections and 774 deaths between 2002 and 2003 (Li et al., 2005; Lau et al., 2010; van Boheemen et al., 2012). Recent surveillance data suggests that camel is a reservoir of MERS-CoV capable of direct transmission to humans, as evidenced by significantly higher seropositivity in dromedary camel exposed individuals, compared to the rest of the study population (Alagaili et al., 2014; Mohd et al., 2016). The extensive spread of the virus in camels in the Middle East and Africa, and their close contact to human populations (Mohd et al., 2016), elevates the pandemic potentials of MERS-CoV.

Recently, virological diagnostic techniques have been widely investigated for detecting MERS-CoV, including real-time polymerase chain reaction (PCR), reverse transcription (RT)-PCR, and quantitative real-time (qRT) PCR (Abd El Wahed et al., 2013; Bhadra et al., 2015). These protocols allow for *in vitro* amplification of specific gene, thus enabling an accurate diagnosis of clinical isolates from patients. Indeed, a sensitive molecular detection for MERS-CoV was achieved by using the upstream of the envelope E (upE) gene and open reading frame (ORF) 1a (Memish et al., 2014; Shirato et al., 2014). Nevertheless, according to the 2013 WHO announcement for MERS-CoV positive detection ^2^, it is still necessary to develop as many specific genetic targets as possible, to allow reliable MERS-CoV diagnoses.

The loop-mediated isothermal amplification (LAMP) method, which amplifies the specific nucleic acid exponentially in a constant temperature environment, has been considered for the rapid detection of viral specific genes in recent years (Kaneko et al., 2005; Iwata et al., 2006; Cai et al., 2008; Huang et al., 2012). LAMP has especially attracted considerable interest since it is a potentially comparable to the popular detection methods such as conventional PCR and real-time PCR, in terms of specificity and sensitivity. For example, *Salmonella* spp., *Escherichia coli, Staphylococcus aureus* and other pathogenic bacteria genome markers have been used for the LAMP-based amplification assay (Aoi et al., 2006; Arunrut et al., 2016). LAMP protocol can also be applied for the detection of RNA viruses, including the SARS corona virus (Hong et al., 2004), influenza viruses (Poon et al., 2005), and other infectious respiratory viruses, by merging the reverse transcription steps into the LAMP processes (Parida et al., 2005). To date, RT-loop-mediated isothermal amplification (RT-LAMP) assay for detection of MERS-CoV RNA, targets within the ORF 1a and ORF1b genes and upstream of the E gene (Bhadra et al., 2015). The researchers are able to improve their RT-LAMP by monitoring the real-time fluorescent signal using the lab-scale PCR machine, but are still limited by the need for gel electrophoresis imaging system, expensive thermal cyclers, and the time-consuming technique.

**FIGURE 1:**
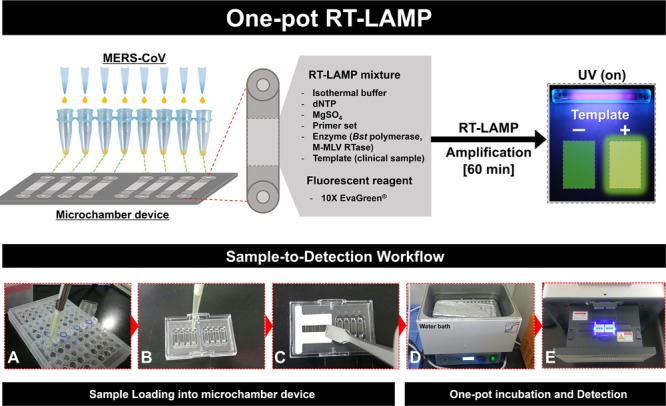
**Schematic illustration of the entire one-pot RT-loop-mediated isothermal amplification (RT-LAMP) workflow, “Sample-to-Detection.” (A–C)**: Sample preparation and loading procedures, **(D)** and **(E)**: One-pot incubation in water bath and loop-mediated isothermal amplification (LAMP) signal read-out analysis.

In this present study, a one-pot RT-LAMP assay has been demonstrated for the specific detection of MERS-CoV nucleocapsid (N) gene that completes a one-pot reaction, “Sample-to-Detection,” to carry out the entire diagnostic protocol within 60 min (see **Figure 1**). Moreover, isothermally amplified specific products can be detected by fluorescence under ultra-violet light using field-deployable microchamber devices, leading to the identification of as few as 0.4 RNA copies of MERS-CoV. Our approach is also potentially applicable for Point of Care Testing (POCT) devices, as a sample-to-detection module in an automatic detection manner.

## Materials and Methods

### Virus RNA Extractions

The MERS-CoV Korean isolate (MERS-CoV/KOR/KNIH/002_05_2015, Genebank accession no. KT029139) was kindly provided by the Korea Centers for Disease Control and Prevention (KCDC). MERS-CoV was propagated and titrated in Vero cells. The virus was titrated by the method of plaque forming unit (PFU). In a 6-well plate, 10-fold serially diluted viruses were used to infect the Vero cells and the mixture of agarose media which contains 2x Eagle minimal essential medium (EMEM; Lonza) with 5% fetal calf serum (FCS) (Gibco), antibiotics (1% penicillin/streptomycin), and 1.8% low-melting agarose was overlayed. Then, the plate was incubated at 37°C/5% CO_2_ for 4 days. Cells were visualized after fixing with 10% neutral buffered formaldehyde and staining with 0.5% crystal violet solution for 30 min. All work with MERS-CoV was conducted in Enhanced BioSafety Level 3 (BL-3+) facility approved by the Korea Center for Diseases Control at Chungbuk National University.

### Template dsDNA Preparation

Viral RNA was extracted from 200 μL of the viruses using the QIAampViral RNA Mini kit (QIAGEN, Valencia, California), and dissolved in 50 μl of eluent. After extraction, RNA was stored at -70°C until used. RNA samples were amplified by RT-PCR using TOPscript^TM^ One-step RT PCR Kit (Enzynomics, Daejeon, Korea) and specific primers (Forward: MERcv-sF_GAGCTTAGGCTCTTTAGTAAG, and Reverse: MERcv-sR_ TTTTTTTTTTTTGCAAATCATCTAATTAGCCTAA) for N gene of MERS-CoV (**Table 1**). The reaction condition for reverse transcription was set at 42°C for 30 min and 95°C for 10 min, followed by 40 cycles of 95°C for 30 s, 60°C for 30 s, and 72°C for 30 s, and then to final elongation at 72°C for 5 min. Total 5 μl of PCR products were analyzed on 2% agarose gel electrophoresis in Tris-buffer, and target bands (780 bp; 28561–29340 bp, see Supplementary Figure S1) were optimized by staining with ethidium bromide.

**Table 1:** One-pot reverse transcriptional loop-mediated isothermal amplification (RT-LAMP) primers for the detection of Middle East Respiratory Syndrome coronavirus (MERS-CoV).

P1_Primers	Sequence (5′–3′)	Position^a^
MERcv-F3	GGAATGGAATTAAGCAACTGGC	28828–28849
MERcv-B3	CGCGAATTGTGTAACAATAGCT	28997–29018
^b^MERcv-FIP	ACAGCCCGGAATGGGAG-GTGGTACTTCTACTACACTGGAA	28896–28912+28856–28878
^c^MERcv-BIP	TAAGGATGGCATCGTTTGGGT-TCATTGTTAGGGTTCCGCG	28913–28933+28975–28993
MERcv-LF	TGCTGCTTCGGGTCCAG	28879–28895
MERcv-LB	GCGCCACTGATGCTCCTTC	28945–28963
^d^MERcv-sF	GAGCTTAGGCTCTTTAGTAAG	28500–28520
^e^MERcv-sR	TTTTTTTTTTTTGCAAATCATCTAATTAGCCTAA	30067–30100

*^a^The MERS-CoV Korean isolate (MERS-CoV/KOR/KNIH/002_05_2015, Genebank accession no. KT029139) was used as the reference sequence.*

*^b,c^ Forward Inner Primer (FIP-1) consists of the F2 at the 3′ end that is complementary to the F2c, and the same nucleotide sequence as the F1c at the 5′ end. Backward Inner Primer (BIP-1) consists of the B2 that is complementary to the B2c region, and the same nucleotide sequence as the B1c at the 5′ end.*

*^d,e^The sF and sR was used for the specific amplification of nucleocapsid (N) gene from MERS-CoV genome.*

### Primer Design for RT-LAMP

To design RT-LAMP primer sets for detection of MERS-CoV, the sequence of nucleocapsid protein (N protein) of MERS-CoV Korean isolate (KT029139, 2015) were compared with that of other MERS-CoV isolated from South Korea (KT374050, 2015; KU308549, 2015; KX034094, 2015), Netherlands (JX869059, 2012), Saudi Arabia (KJ156944, 2013; KM027255, 2014; KT806054, 2015; KU851863, 2015), England (KM015348, 2013), Egypt (KJ477102, 2014), Qatar (KF961221, 2014), and United States (KJ813439, 2014). The conserved part was selected and used as the template of RT-LAMP.

To find out an efficient primer set, two sets of specific RT-LAMP primers were designed using PrimerExplorer V4 software program^3^ based on the published sequence of N gene. Also, two primer sets were added to the T4 space sequence (5′-TTTT-3′) to forward and backward inner primer (FIP, BIP). The detailed information of each primer sets used in this study is listed and labeled in Supplementary Table S1.

### Determination of Optimal RT-LAMP Primer Sets

For optimization of efficient RT-LAMP primer sets, four primer sets were used for isothermal amplification using dsDNA template for N gene, partial amplified product of MERS-CoV genome (780 bp; 28561–29340 bp). LAMP reaction mixture contained 2.5 μL 10X isothermal amplification buffer [1X contained 20 mM Tris-HCl, 10 mM (NH_4_)_2_SO_4_, 150 mM KCl, 2 mM MgSO_4_, 0.1% Tween^®^ 20], 1 μL each outer primer (F3, B3; 5 μM), inner primer (FIP, BIP; 20 μM), and loop primer (LF, LB; 5 μM), 2 μL 10 mM dNTP, 1.5 μL 100 mM MgSO_4_ (total 8 mM), and up to 23 μL distilled water. After addition of 1 μL dsDNA template, the mixture was heated at 95°C for 5 min, and then cooled to room temperature for 5 min. Next, 1 μL (eight units) of *Bst* polymerase was added to the mixture, LAMP reaction was performed at 63°C for 60 min, and then heated to 80°C for 10 min for termination. The results of LAMP were analyzed using agarose gel electrophoresis. Also, changes were visually observed based on color change (positive: Green, negative: Orange), and decrease in fluorescence of SYBR^®^ green I (Thermo Fisher Scientific, Waltham, MA, USA) was examined under UV light (Gel Doc^TM^ EZ Gel Documentation, Bio-Rad, Hercules, CA USA).

### Optimization of RT- LAMP Conditions

For the single step RT-LAMP detection of MERS-CoV, it is very important to optimize the M-MLV reverse transcriptase units as well as *Bst* polymerase. The RT-LAMP reactions were performed at conditions of M-MLV 100 and 200 units, and *Bst* polymerase 1, 2, 4, 6, and 8 units. To determine the optimal reaction time, reactions were performed at 63°C for 30, 40, 50, 60, 90, and 120 min. On completion of the reaction, the amplified results were detected by color change, using 2 μL of 1000X SYBR^®^ green I (Thermo Fisher Scientific, Waltham, MA, USA). After addition of SYBR-green I, samples that turned yellowish green were considered positive. By contrast, when color remained orange, the reaction was assumed to be negative. All PCR/LAMP products were detected by 2% agarose gel electrophoresis and the fluorescence was visualized under UV light.

### Sensitivity Comparison of Conventional RT-PCR, Real-Time qRT-PCR and RT-LAMP

RNA samples were serially diluted 10-fold to conduct the sensitivity comparison between RT-LAMP, conventional RT-PCR, and quantitative (q) RT-PCR. Using the diluted RNA, the RT-LAMP reaction was performed at 63°C for 60 min, and terminated by heating to 80°C for 10 min. The verified primer set, Primer 1 (P1) was used for RT-LAMP reaction under the following conditions: 1 μL each of outer primer (F3, B3; 5 μM), inner primer (FIP, BIP; 20 μM), loop primer (LF, LB; 5 μM), and 1 μL of diluted RNA samples ranging from 4 × 10^3^ to 4 × 10^-1^ infectious viral genome copies/μL. Conventional RT-PCR was performed using 1 μL outer primer (F3, B3, each 5 pmol/μL) and equal RNA template (similar to RT-LAMP reaction) with TOPscript^TM^ One-step RT PCR Kit (Enzynomics, Daejeon, KOREA ^4^) under the following conditions: reverse transcription at 42°C for 30 min, initial denaturation at 95°C for 10 min, 35 cycles of three steps; denaturation at 95°C for 30 s, annealing at 60°C for 30 s, elongation at 72°C for 30 s, and final elongation at 72°C for 5 min. qRT-PCR was also performed using 1 μL outer primer (F3, B3; 5 μM) and equal template (as used for RT-LAMP reaction) with TOPreal^TM^ One-step RT qPCR Kit (SYBR Green, low Rox) (Enzynomics, Daejeon, KOREA^5^) under the same conditions conducted for conventional RT-PCR mentioned above. The results of RT-LAMP and RT-PCR were identified using agarose gel electrophoresis. qRT-PCR was performed and identified by using real-time machine, CFX96 Touch^TM^ Real-Time PCR Detection System (Bio-Rad, Hercules, CA, USA).

### One-Pot RT-LAMP in Microchamber

In order to utilize EvaGreen in one-pot RT-LAMP experiment, the sensitivity of EvaGreen^®^ (Biotium Inc., USA) were compared with SYBR^®^ Green I (Thermo Fisher Scientific, Waltham, MA, USA) for each concentration (non-contained, 1, 2, 5, 10, 20, 50, 100, 200, 500 and 1000X). Polymer based microchamber devices were purchased from Dongwoo Science Co., Ltd (Republic of Korea). Schematic illustration (**Figures 1A–C**) shows the entire experimental concept for the one-pot RT-LAMP protocol, “Sample-to-Detection workflow.” Assays were performed in a single unit of microchamber, using final volume 10 μL, consisting of 0.5 μL each outer primer (F3, B3; 5 μM), inner primer (FIP, BIP; 20 μM), loop primer (LF, LB; 5 μM) and 1 μL of RNA template (4 × 10^3^ to 4 × 10^-1^ infectious viral genome copies/μL). Each microchamber was protected with a thin cover film during the isothermal reaction period. The RT-LAMP was performed in a water bath, by maintaining the temperature at 63°C for 60 min. The fluorescent results of microchamber amplification were visualized by UV-image capture process, using the GENECHECKER^TM^ Ultra-Fast PCR System (Dongwoo Science Co., Ltd, republic of Korea). The RT-LAMP products were also confirmed by 2% agarose gel electrophoresis.

### Specificity of MERS-CoV RT-LAMP

The specificity of RT-LAMP reaction for MERS-CoV was identified using the one-pot RT-LAMP reaction and comparing to other acute respiratory disease viruses: the human coronavirus (HCoV)-229E, human metapneumovirus (HMPV), and the influenza viruses – A/California/04/2009 (H1N1pdm), A/Perth/16/2009 (H3N2), B/Brisbane/60/2008 (Victoria lineage), and B/Phuket/3073/2013 (Yamagata lineage). HCoV-229E and HMPV were obtained from the Chungbuk National University Hospital in South Korea. The influenza virus, A/California/04/2009 (H1N1pdm), was kindly provided by the St. Jude Children’s Research Hospital; A/Perth/16/2009 (H3N2), B/Brisbane/60/2008 (Victoria lineage), and B/Phuket/3073/2013 (Yamagata lineage) were kindly provided by Green Cross Corp. (Korea). Specific primer sets to identify existence of virus genome were custom-designated, as listed in Supplementary Table S2. Total RNA was extracted from samples using the RNeasy prep-kit (QIAGEN, Valencia, California) and RT-PCR was conducted using TOPscript^TM^ One-step RT PCR Kit (Enzynomics, Daejeon, Korea) under the following conditions: reverse transcription at 42°C for 30 min, initial denaturation at 95°C for 10 min, 35 cycles of three steps; denaturation at 95°C for 30 s, annealing at 60°C for 30 s, elongation at 72°C for 30 s, and final elongation at 72°C for 5 min. The results of RT-PCR were identified by using agarose gel electrophoresis, as described in the previous section. The extracted RNA of all viruses and MERS-CoV were used as templates for one-pot RT-LAMP with 10X EvaGreen^®^ (Biotium Inc., Hayward, California, USA).

### Ethics Statement

Clinical samples were collected from the Chungbuk National University Hospital, South Korea. Nasopharyngeal aspirate samples collected in accordance with the approved guidelines and relevant regulations. All subjects provided their written informed consent prior to participating in the study. The Ethics Committee and IRB (Institutional Review Board) of Chungbuk National University Hospital approved all experimental procedures. All experiments were performed in accordance with the approved guidelines.

## Results

### LAMP Target and Primer Efficiency

From May 2015 to July 2015, a total of 186 cases, including 36 deaths, have been reported in South Korea. The MERS-CoV (MERS-CoV/KOR/KNIH/002_05_2015) Korean isolate (GenBank KT029139) was propagated and titrated using Vero cells. Total RNA of MERS-CoV was collected, and the synthesis of complementary DNA (cDNA) from a viral RNA template, via reverse transcriptase (M-MLV), was performed. Detailed information about RNA/cDNA preparation was referenced in the supplementary information. As shown in Supplementary Figure S1, 780 bp products were specifically amplified, indicating the successful retrievals of viral nucleocapsid gene sequence.

**FIGURE 2:**
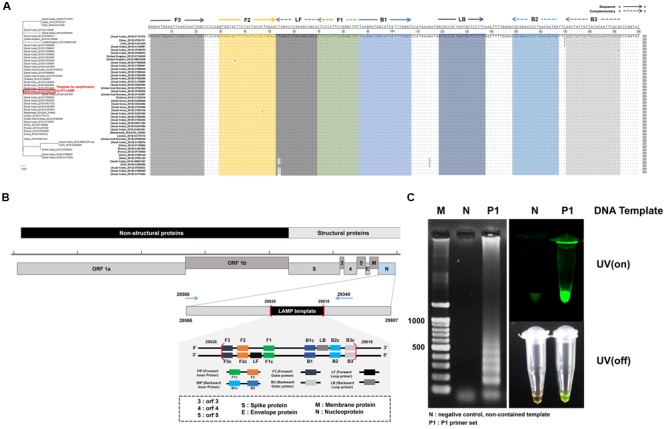
**Middle East Respiratory Syndrome coronavirus (MERS-CoV) nucleocapsid (N) protein gene sequences and RT-LAMP primer design. (A)** The sequences alignment was conducted using the sequence of the 2015 Korean isolate (KT029139). Phylogenetic analysis based on the nucleotide sequences corresponding to the full-length of N gene. The GenBank accession numbers of recent virus strains are in the brackets. The essential LAMP primers of F3, F2, F1, B2, B3, and loop primers of LF and LB were located and highlighted. **(B)** FIP (Forward Inner Primer) contains FIc (c: complementary) and F2. BIP (Backward Inner Primer) contains the sequence (B1c) complementary to B1. Forward and backward loop primer (LF and LB, respectively) were designed to accelerate the amplification reaction. LAMP target sequence used for primer design are shown in the box (position 28828 ∼ 29018). **(C)** LAMP efficiency by primer set 1 (P1). All sequence information is listed in **Table 1**. The positive LAMP amplification is clearly realized by Primer set 1 (P1). Lane M: 100 bp DNA ladders, lane N: negative control, non-template contained, lane P1: the results amplified by the primer set 1 (P1).

It is noteworthy that the number of MERS-CoV sequences reported in GeneBank is proportionally increasing. Forty three representative MERS-CoV sequences deposited during outbreaks in 2012–2015 were aligned with the Korean isolate (Accession No. KT029139). The LAMP primer sets were considered based on the conserved regions of the N gene sequence, which exhibits only thirteen nucleotide mismatches among the broadly ranged strains at position 28828 to 29018, as shown in **Figure 2A**. When we focused on the LAMP primer sequences area, there were seven nucleotides mismatches. Especially, six mutations were observed in Saudi Arabia strains (KF600613, 2012; KT806048, 2015; KU851859, 2015; KM027257, 2014; KT806055, 2015; KU710264, 2015). One leftover sequence was found in USA isolate (KJ829365, 2014).

Loop-mediated isothermal amplification reaction was initially evaluated with four candidate groups of six essential LAMP primers that are available to recognize the distinct regions on the target N gene (F1, F2, F3, B1, B2, and B3). Two additional loop primers (LF and LB) were used to enhance the LAMP reaction. The standardized dsDNA (1 ng/μL of MERS-CoV viral N gene) was used. The sequences of the primers and their locations are indicated in **Figure 2B**, and **Table 1**. According to the previous study of LAMP assays (Kuboki et al., 2003; Parida et al., 2008), typical thymine spacers were used to enhance LAMP efficiency by assisting the loop formation. Hwang et al. (Yang et al., 2010) investigated the effects of LAMP amplification associated by inserting T-bases into the FIP and BIP, which suggested that the LAMP primer containing T4 linker sequences would be greater than the conventional PCR method. However, in our experiment, the T4 linker didn’t seem to affect gene amplification, and its use was rather a failure in LAMP reaction. Supplementary Figure S2 shows the experimental results for the LAMP amplification depending on the primer configurations (P1, P1-T4, P2, and P2-T4). The experimental verification of the LAMP primer performance was critical for the successful amplification. Agarose gel electrophoresis and SYBR green I visible evidence (UV on/off) show that the P1 primer compositions contain the typical LAMP signature, as shown in **Figure 2C**. Moreover, there is only one mismatch in the essential F2, B2, and B3 regions, compared to the individual sequences of the other MERS-CoV (**Figure 2A**). Thus, the Primer 1 set (P1) was selected for further investigations. We believe that the LAMP primer set utilized in this study is available to detect MERS-CoV N gene as a universal element in LAMP assay.

### Optimized RT-LAMP Reaction

In order to determine the optimum units (U) of enzyme (M-MLV, Moloney Murine Leukemia Virus reverse transcriptase and *Bst* polymerase) and the reaction time, the optimal conditions of RT-LAMP reaction were applied, using MERS-CoV RNA as the template. Especially, the M-MLV reverse transcriptase (RTase) shows the high thermostable activity that can synthesize cDNA up to 65°C (Arezi and Hogrefe, 2009). All RT-LAMP reactions were therefore carried out at a temperature of 63°C for 60 min.

**FIGURE 3:**
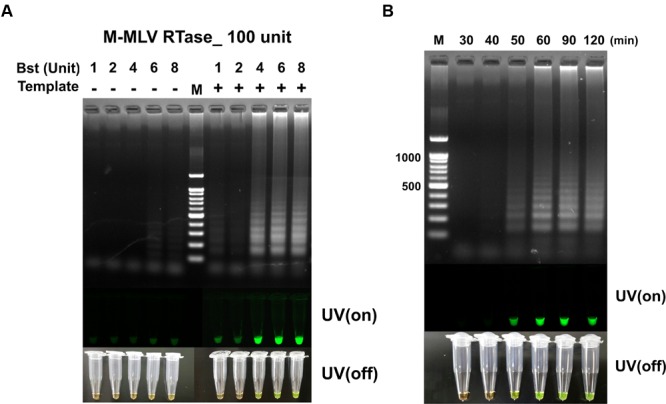
**(A)** The RT-LAMP detection results based on different ratios of *Bst* polymerase and M-MLV reverse transcriptase concentration (100 unit). Concentration of *Bst* polymerse is 1, 2, 4, 6, and 8 units. **(B)** Effect of reaction time for RT-LAMP reaction. DNA ladder-like pattern was confirmed by 2% agarose gel electrophoresis. LAMP products detected by adding 1,000X SYBR-green I when the reaction was completed (UV/on and off). Land M: 100 bp DNA ladders; - depicts tube used as negative control, non-template; + indicates the results with template.

The unit of M-MLV RTase was fixed at 100 U, the RT-LAMP reaction at the dosage of *Bst* polymerase increased from 4 to 8 U, without non-template self-constructions. However, the M-MLV RTase 200 U tends to generate non-template self-constructions at all *Bst* polymerase conditions (Supplementary Figure S3). It should be noted that a significant difference was observed in RT-LAMP reaction, depending on the dosage of reverse transcriptase. The amount of reverse transcriptase can be a critical element for a successful LAMP amplification for RNA templates. As a result, 100 U M-MLV RTase and 4 U *Bst* polymerase were selected as the optimum RT-LAMP amplification of MERS-CoV N gene, achieving sufficient visible change under UV on/off (**Figure 3A**).

The RT-LAMP with different reaction times of 30, 40, 50, 60, 90, and 120 min for detecting MERS-CoV N are shown in **Figure 2B**. The clearest LAMP products were observed as early as 50 min at 63°C. However, the reaction time was set up at 60 min to ensure a positive reaction with low template concentration. Our results indicated that the speed of the reaction was significantly improved by incorporating M-MLV RTase (100 U) in the reaction. It was also clearly observed that the RT-LAMP amplification can be detected in visible changes with SYBR-green I.

### Comparison in Sensitivity for Optimized RT-LAMP

**FIGURE 4:**
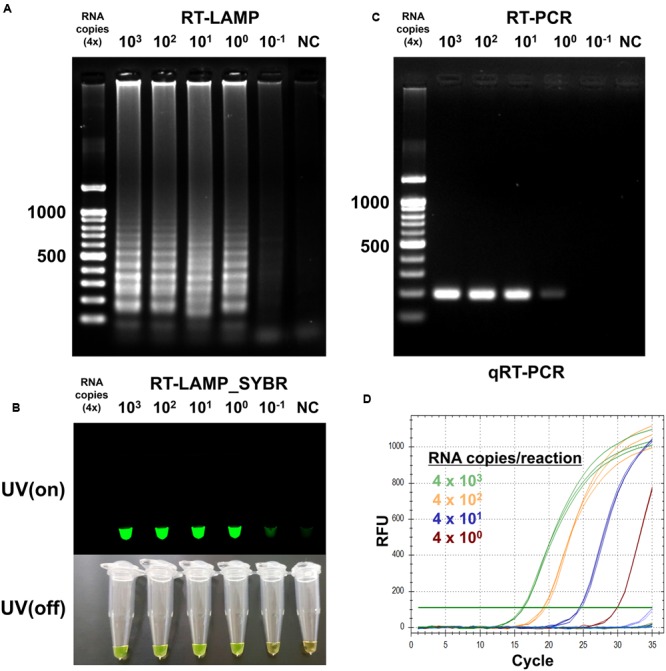
**Sensitivity of the RT-LAMP in comparision with conventional methods.** To evaluate sensitivity of the RT-LAMP, MERS-CoV RNA was serially diluted in the range 4 × 10^3^ to 4 × 10^-1^/μL. The RT-LAMP reaction was performed using 1 μL of diluted samples with primer set 1 (see **Table 1**). The reaction result was observed by **(A)** DNA gel electrophoresis, **(B)** UV and naked eye. For comparison, **(C)** conventional RT-PCR and **(D)** real-time qRT-PCR were performed, using equal amount of RNA used for RT-LAMP reaction. Agarose gel analysis for qRT-PCR was confirmed as shown in Supplementary Figure S4. NC, negative control.

To determine detection limit of the optimized RT-LAMP method, we sought to amplify the endpoint-diluted viral RNA extracted from Korean isolated MERS-CoV grown in Vero cells. Total RNA was purified from 200 μL of the virus, which titered to 1 × 10^6^ PFU/mL; the concentration of viral RNA was found to be 4 × 10^3^ infectious viral genome copies/μL. To perform the RT-LAMP as described and optimized above, we used 1 μL of endpoint diluted RNA samples, ranging from 4 × 10^3^ to 4 × 10^-1^ RNA copies per reaction. For comparison, conventional quantitative RT-PCR and reverse transcription PCR was performed using equally diluted RNA samples for RT-LAMP with F3 and B3 primers. The positive reaction was clearly observed in the 4 RNA copies of MERS-CoV RNA sample in the optimized RT-LAMP reaction, using 2% agarose gel electrophoresis, UV and naked eye (**Figures 4A,B**). Although both the conventional RT-PCR and qRT-PCR detected up to the 4 RNA copies of MERS-CoV RNA sample, the RT-LAMP method reduced the detection time up to 100 min, as compared to the conventional methods (**Figures 4C,D**). Significantly, with RT-LAMP, it was possible to detect the presence of viral RNA genome without a requirement for a reverse transcription step [total reaction time for RT-LAMP: 60 min, **Figure 3B**]. The overall result demonstrates that the sensitivity of the RT-LAMP optimized via this study targeting N gene of MERS-CoV is a minimum of four viral RNA copies, implying high sensitivity as compared to the conventional RT-PCR and qRT-PCR methods.

### One-Pot RT-LAMP Reaction in a Microchamber

Optimum RT-LAMP reaction was evaluated to detect MERS-CoV by using the N gene-specific LAMP primer set (set P1, see Supplementary Figures S2 and S6A). All signal read-outs were distinguished after amplification between positive and negative results in end-point analysis by adding 2 μL of SYBR-green I dye to the LAMP reactants. Although the SYBR-green I based end-point signal read-out is more robust and effective than the fluorescent and naked-eye LAMP detection, the difficulty exists in applying in one-pot analysis, since the highly concentrated SYBR-green I dye is a strong inhibitor of DNA polymerase activity during the thermal cycling process. In order to study the effect of the SYBR-green concentration on one-pot RT-LAMP reaction, different concentrations were introduced into the mixture prior to the RT-LAMP amplification. As presented in Supplementary Figure S5, the electrophoresis gel data clearly confirm that successful RT-LAMP amplification achieved in the range 0 ∼ 50X concentrated SYBR-green. However, as expected, we found that the fluorescent SYBR-green I signal observed in the highly concentrated solutions (100 ∼ 1000X) failed to represent any significant correlation between the LAMP-amplification and positive response. Even the signal difference between positive and negative samples was not obvious at maximum SYBR-green I concentration (50X, not inhibited to LAMP amplification). This suggests that an alternative detection method should be adopted to realize the one-pot RT-LAMP reaction.

**FIGURE 5:**
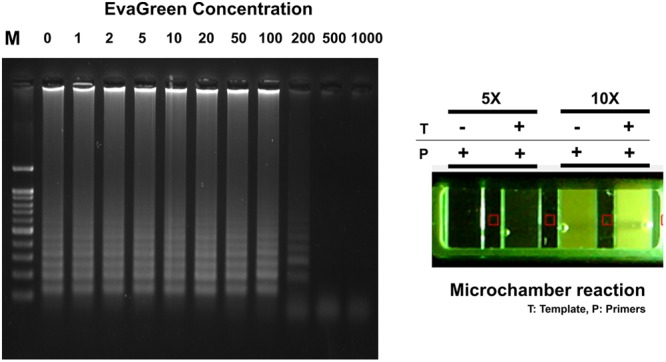
**EvaGreen activity for RT-LAMP.** Resultant microchamber devices following the loop-mediated isothermal amplification (LAMP) reaction with EvaGreen (5 and 10X) under UV (On). DNA ladder-like LAMP amplification pattern was confirmed by 2% agarose gel electrophoresis. T: template; P: LAMP-primers. The fluorescence backgrounds were shown as red-squares.

Typically, EvaGreen emits a weak fluorescent signal in the presence of DNA primers or templates (ssDNA or cDNA), but emits strongly upon binding to dsDNA as a dsDNA intercalator (Mao et al., 2007). The electrophoresis agarose gel data clearly confirm that successful RT-LAMP amplification achieved in the range 0 ∼ 100X concentrated EvaGreen. This result also provides the high resolution fluorescent sensitivity that allows us to evaluate the success of LAMP reaction at a low concentration (10X) (**Figure 5**). Moreover, it exhibits less amplification inhibition (not inhibiting when used 1 ∼ 100X), which makes it possible to be present throughout the entire LAMP reaction, and therefore may be applicable for one-pot RT-LAMP platform.

The one-pot RT-LAMP assay was next conducted in a commercialized polymer-microchamber, where MERS-CoV RNA template, freely mixed in an amplification buffer solution containing LAMP primers, *Bst* polymerase, M-MLV RTase, and EvaGreen (10X) were initially embedded. In addition, the commercial microchamber experiment enables the low consumption of expensive reagents. A final volume of 10 μL RT-LAMP mixture was introduced into the single unit of microchamber of a polymer device. After 60 min of incubating at 63°C in a conventional water-bath, RT-LAMP reaction with 10X EvaGreen clearly presented to be positive for MERS-CoV (**Figure 5**).

**FIGURE 6:**
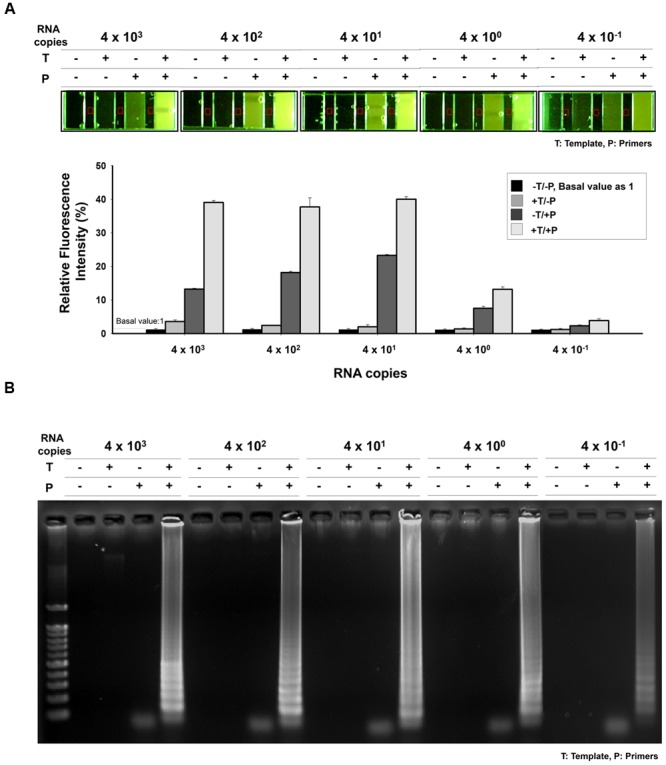
**Sensitivity of one-pot RT-LAMP. (A)** 10X EvaGreen was initially mixed with RT-LAMP reagents, and the reaction was performed and visualized in microchamber. The purified MERS-CoV RNA was serially diluted in the range 4 × 10^3^–4 × 10^-1^/μL. Relative fluorescent signals were analyzed by using ImageJ software. The relative intensity of fluorescence for positive amplification was normalized to the controls (-Template/-Primer). Red square indicates background fluorescence. **(B)** Agarose gel electrophoresis confirmation indicated that one-pot RT-LAMP can detect MERS-CoV as few as 0.4 RNA copies. DNA ladder-like pattern was confirmed by 2% agarose gel electrophoresis. T, template; P, primer.

The sensitivity of the one-pot RT-LAMP assay for MERS-CoV was determined by testing serial RNA, ranging from 4 × 10^3^ to 4 × 10^-1^ infectious viral genome copies/μL. The RT-LAMP assay could detect as few as 0.4 RNA copies, as evidenced from the microchamber device (**Figure 6A**) and agarose gel electrophoresis results (**Figure 6B**). In order to analyze the relative fluorescent signals in the microchamber, intensity measurement and image processes were performed with ImageJ program. The background fluorescence (red square) was subtracted and fluorescence intensity for positive amplification was normalized to the controls (-Template/-Primer). Relative fluorescence intensities of positive amplification (+Template, +Primer) indicated that one-pot RT-LAMP in microchamber showed a 10-fold improvement over the previous non-microchameber RT-LAMP assay (see **Figure 4A**, PCR-tube RT-LAMP reaction), that might be due to the microfabricated device benefits such as small-volume operation and high heat transfer/maintenance coefficients (Zhang and Xing, 2007).

### Specificity of One-Pot RT-LAMP Reaction for MERS-CoV

**FIGURE 7:**
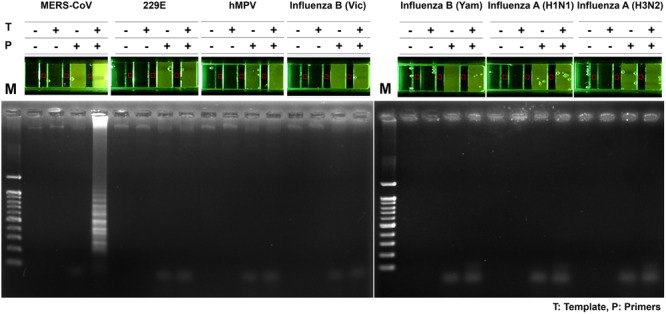
**Specificity of one-pot RT-LAMP for MERS-CoV.** RNA samples of MERS-CoV and the other respiratory pathogens were used for specificity evaluation of one-pot RT-LAMP system for MERS-CoV detection; fluorescence was visualized by 10X EvaGreen in microchamber. Each set of lane indicates a specific virus: lane 1, MERS-CoV Korean isolate; lane 2, Human coronavirus (HCoV)-229E; lane 3, Human metapneumovirus (HMPV); lane 4, B/Brisbane/60/2008 (Victoria lineage); lane 5, B/Phuket/3073/2013 (Yamagata lineage); lane 6, Influenza A virus (A/California/04/2009, H1N1); lane 7, influenza A virus (A/Perth/16/2009, H3N2). M, 100 bp DNA ladder.

To assess the specificity of the MERS-CoV optimized RT-LAMP in other acute respiratory disease viruses (including influenza type A, H1N1, H3N2, and type B) or clinical samples (including human coronavirus 229E and human metapneumovirus obtained from patients) previously validated by PCR and sequence analysis (Supplementary Table S2), total RNA was extracted and evaluated by the optimized MERS-CoV RT-LAMP. In addition, conventional RT-PCR of the respiratory virus RNA panel was performed, using their specific and customized primer sets, to verify the respiratory-related viral samples (Supplementary Table S2). As shown in gel electrophoresis and one-pot visualization by EvaGreen (**Figure 7**), the MERS-CoV RNA was the only positive sample for the optimized RT-LAMP, while the other viruses were positive for conventional RT-PCR using their specific primer sets (see Supplementary Figure S6).

## Discussion

Middle East Respiratory Syndrome coronavirus diagnosis was initially performed using a conventional real-time RT-PCR assay. In order to carry out an accurate diagnosis, at least two different genomic targets are necessary. The study by Corman et al. (2012, 2014) reported high sensitivity by targeting ORF1a and upE with specific primer sets, combining with reverse transcription. Recently, several methods based on LAMP have been reported for specific MERS-CoV. Bhadra et al. (2015) detected the ORF1a, ORF1b and upE using real-time RT-LAMP, finding that one-step strand displacement probes (OSD) could detect 5–50 PFU/ml of MERS-CoV. Shirato et al. (2014) also used RT-LAMP in assessing the nucleocapsid gene, and found that MERS-CoV real-time RT-LAMP enabled detection of LAMP products by observing real-time turbidity, magnesium pyrophosphate precipitation, and SYBR fluorescent signal. Compared to the conventional PCR, the LAMP is a relatively feasible, cost-effective, naked-eye visible and rapid (usually taking 30 ∼ 60 min) method, suggesting that the assay holds greater promise for local on-site diagnosis.

Despite the advantages of the LAMP application, there are still some technical shortcomings, such as false-positive signal read-out, due to the carry-over contamination caused by aerosol during assay manipulations. In addition, end-point detection for observing LAMP signals requires opening of the reaction tube caps; this is problematic when employing to large-scale screening and on-site field application. In this study, the one-pot RT-LAMP assay for detection of MERS-CoV was designed using EvaGreen modified microchamber method for visualizing the resultant signal. SYBR-green and EvaGreen are well known DNA-intercalating dyes for PCR. However, as shown in **Figure 5**, EvaGreen was more suitable to LAMP monitoring because it did not significantly affect to *Bst* DNA polymerization, and therefore it was acceptable to be present throughout the entire one-pot RT-LAMP reaction.

We explored the one-pot application for the detection of LAMP amplicons in the polymer based commercial microchamber device. Molecular analytical systems based on microdevices allow the cartridge-type integration of processes that were previously employed in high through-put DNA amplification, with improved speed and efficiency (Wang et al., 2006). Our experiments conducted with the microchamber demonstrated high sensitivity and specificity comparable to the benchtop PCR results. After loading the one-pot RT-LAMP mixture into a single unit of microchamber, the reaction was performed for 60 min, using a basic water-bath. Finally, the one-pot RT-LAMP in a microchamber displayed sufficient product sensitivity and specificity, which were useful to distinguish between MERS-CoV and other respiratory viruses, such as 229E, MPV, Influenza type B (Yamagata lineage), Influenza type B (Victoria lineage), Influenza type A-H1N1 and Influenza type A-H3N2. The method of this study can be intended to realize the integration into POCT equipment and existing hospital diagnostic system.

**FIGURE 8:**
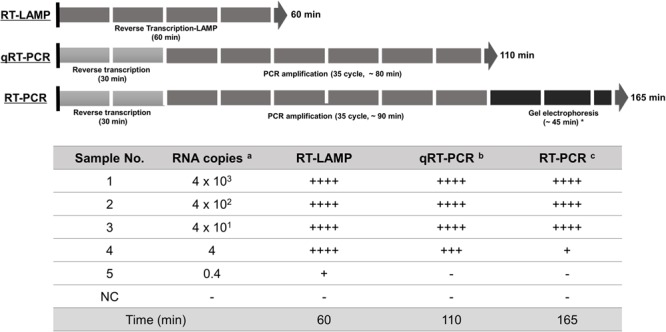
**Summary reaction features for RT-LAMP, real-time qRT-PCR, and RT-PCR assay.** The reaction time of the assays including RT-LAMP, real-time qRT-PCR, and RT-PCR are described using arrow box with duration time for all reactions. Levels of sensitivity of the assays at each dilution point of RNA are validated based on the DNA gel electrophoresis and expressed (see **Figure 4**): ++++, high sensitive; +++, medium-high sensitive; ++, moderate sensitive; +, low sensitive; -, negative. ^a^ 10-fold serially diluted RNA copy number per μl extracted from 1 × 106 PFU/ml of MERS-CoV; ^b^ and ^c^ the reaction time for total experimental procedures was measured based on the manufacturer’s instructions; ^∗^ In our experimental set-up; NC, negative control.

A critical are of diagnosis is undoubtedly the sensitivity to the target. The one-pot RT-LAMP optimized in this study could detect as few as four viral RNA copies, which is comparable to previous reports (Shirato et al., 2014; Bhadra et al., 2015). Also, the optimized RT-LAMP is as sensitive as the conventional RT-PCR and Real-Time qRT-PCR method (**Figure 8**), which is believed to be one of the most sensitive methods developed so far. The N gene of MERS-CoV used as the target for RT-LAMP detection is one of the recommended regions for MERS-CoV detection ^6^. Although the replication mechanism of MERS-CoV has not been fully understood, coronavirus is believed to generate six subgenome-length mRNAs (Zaki et al., 2012; Mailles et al., 2013; Bialek et al., 2014; Memish et al., 2014), all of which contain the N gene region (Corman et al., 2014; Shirato et al., 2014). The subgenomic mRNAs preferably exist within the virally infected cells, and not outside the cells. However, the clinical specimens include not only MERS-CoV virions, but also infected host cells probably containing the subgenomic mRNAs, implying the possible enhancement of RT-LAMP detection targeting the N gene. Thus, N gene could be an attractive target for coronavirus detection.

The infection has flu-like symptoms, and MERS-CoV can be transmitted by patients through respiratory droplets as well as fomites. The recent outbreak of MERS-CoV in South Korea revealed that a rapid and reliable diagnostic assay needs to be urgently developed. Although similar RT-LAMP methods targeted to ORF1a, ORF1b and upE have previously been reported (Abd El Wahed et al., 2013; Bhadra et al., 2015), the optimized method described here, targeting the N gene of MERS-CoV and having a high sensitivity and specificity using the recent Korean isolate, could be an alternative RT-LAMP method for MERS-CoV detection. Furthermore, LAMP primer sets targeting N in our study are acceptable to be employed in one-pot RT-LAMP assay for MERS-CoV, conferring more enriched practical tools for POCT diagnosis. The selectivity and rapidity makes it suitable for large-scale diagnostic tests in emergency.

## Author Contributions

SL and YB contributed equally to this work and performed all experiments. Y-HK, and Y-KC helped conceive the virus specificity study and provided input in writing the manuscript. Y-KC supervised the research and provided BSL-3 Facility. M-SS and J-YA designed the overall experimental concept and revised manuscripts.

## Conflict of Interest Statement

The authors declare that the research was conducted in the absence of any commercial or financial relationships that could be construed as a potential conflict of interest.

The reviewer SF and handling Editor declared their shared affiliation, and the handling Editor states that the process nevertheless met the standards of a fair and objective review.

## Footnotes

1 http://www.who.int/emergencies/mers-cov/en/

2 http://www.who.int/csr/disease/coronavirus_infections/case_definition/en/index.html

3 http://primerexplorer.jp/elamp4.0.0/index.html

4 http://www.enzynomics.com/sub0204/goods/view/cf_code/5/id/84

5 http://www.enzynomics.com/sub0201/goods/view/id/218\#display-zone

6 http://www.who.int/csr/disease/coronavirus_infections/MERS_Lab_recos_16_Sept_2013.pdf
