# Supplementary material for: One-Pot Reverse Transcriptional Loop-Mediated Isothermal Amplification (RT-LAMP) for Detecting MERS-CoV
Source: Front Microbiol. 2017 Jan 9;7:2166. doi: 10.3389/fmicb.2016.02166 (PMC5220095; doi:10.3389/fmicb.2016.02166)
Supplement: Supplementary file 1 [file Data_Sheet_1.docx]

Supplementary Material

One-pot Reverse Transcriptional Loop-mediated Isothermal Amplification (RT-LAMP) for Detecting MERS-CoV

Se Hee Lee ^1, ‡^, Yun Hee Baek ^2, ‡^, Yang-Hoon Kim ^1^, Young-Ki Choi ^2^, Min-Suk Song ^2,^*, and Ji-Young Ahn*^,1^

*** Correspondence:** To whom correspondence should be addressed. Tel: [+82-43-261-2301; Fax: [+82-43-264-9600]; Email: [jyahn@chungbuk.ac.kr], Correspondence may also be addressed. Email: [songminsuk@chungbuk.ac.kr]

# Supplementary Figures and Tables

#
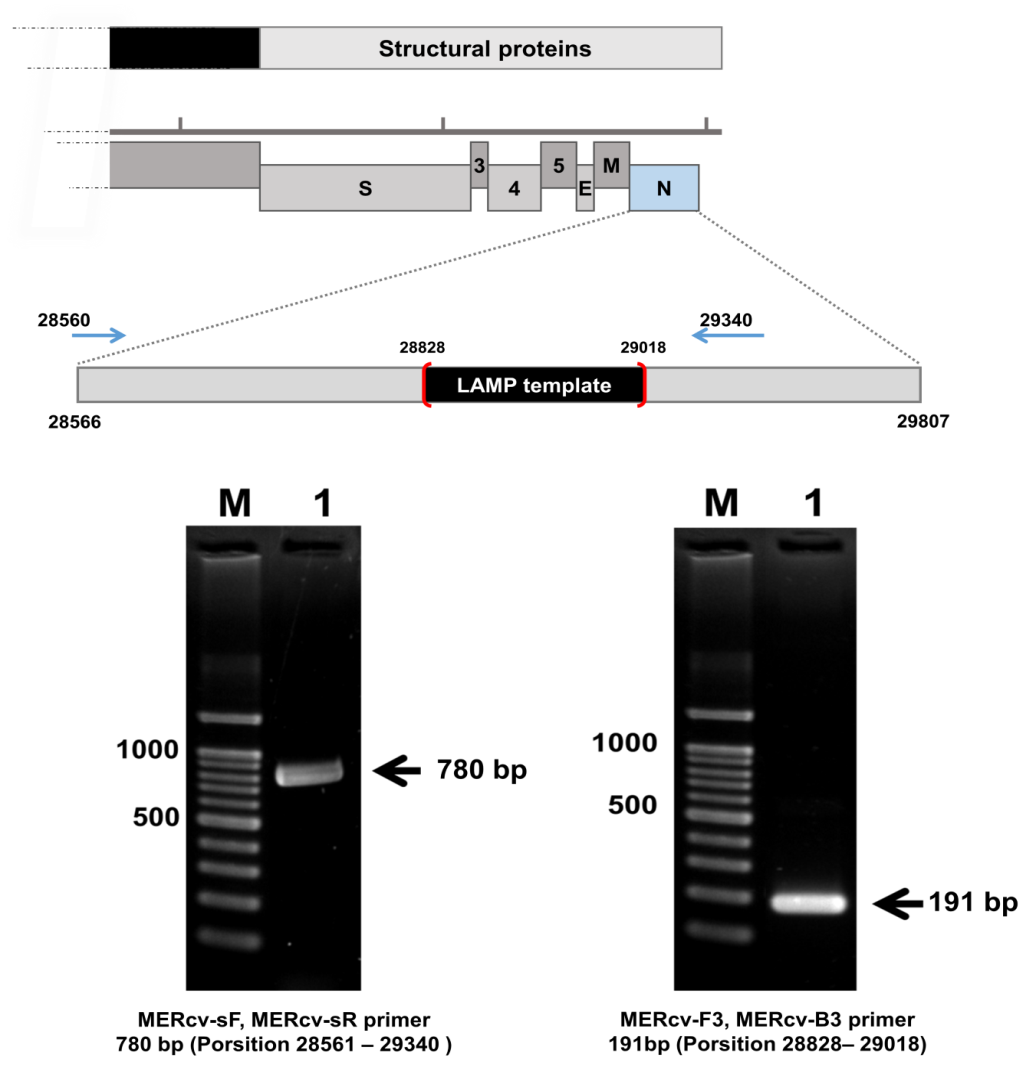


## Figure S1. Amplification of N target using specific primers (780 bp, sF and sR primer) and LAMP target by F3 and B3 primers (191 bp, position 28828 – 29018, see Table 1)


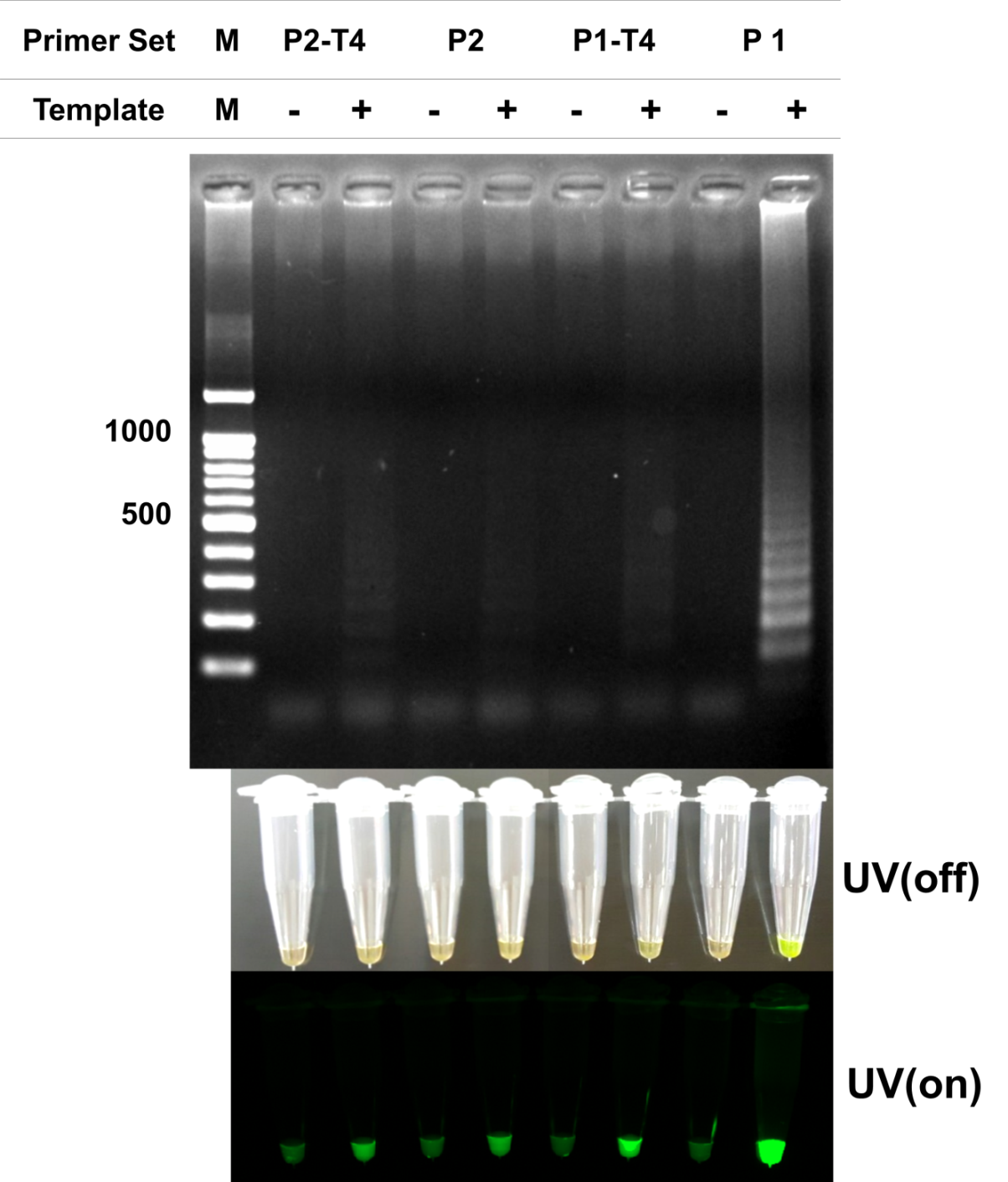


**Figure S2.** LAMP efficiency. The LAMP assay indicated that the positive LAMP amplification was clearly realized by Primer set 1 (P1).


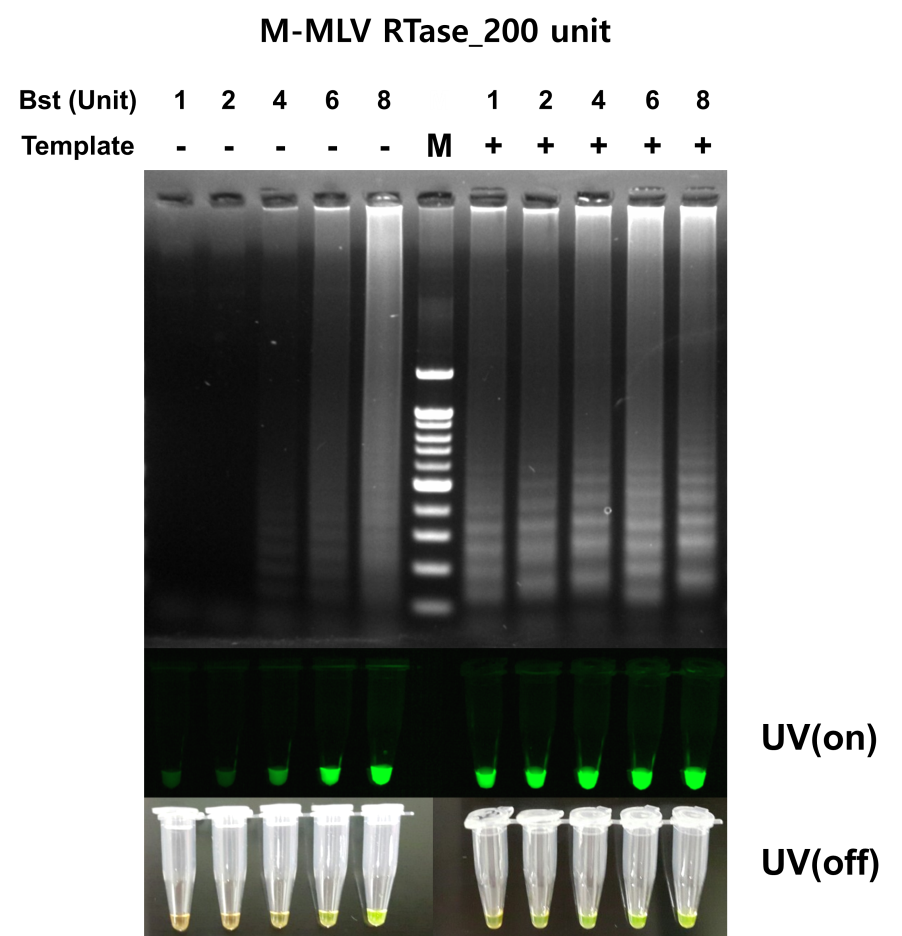


**Figure S3.** The RT-LAMP detection results based on different ratios of *Bst* polymerase and M-MLV reverse transcriptase concentration (200 unit). Concentration of *Bst* polymerse is 1, 2, 4, 6, and 8 units.


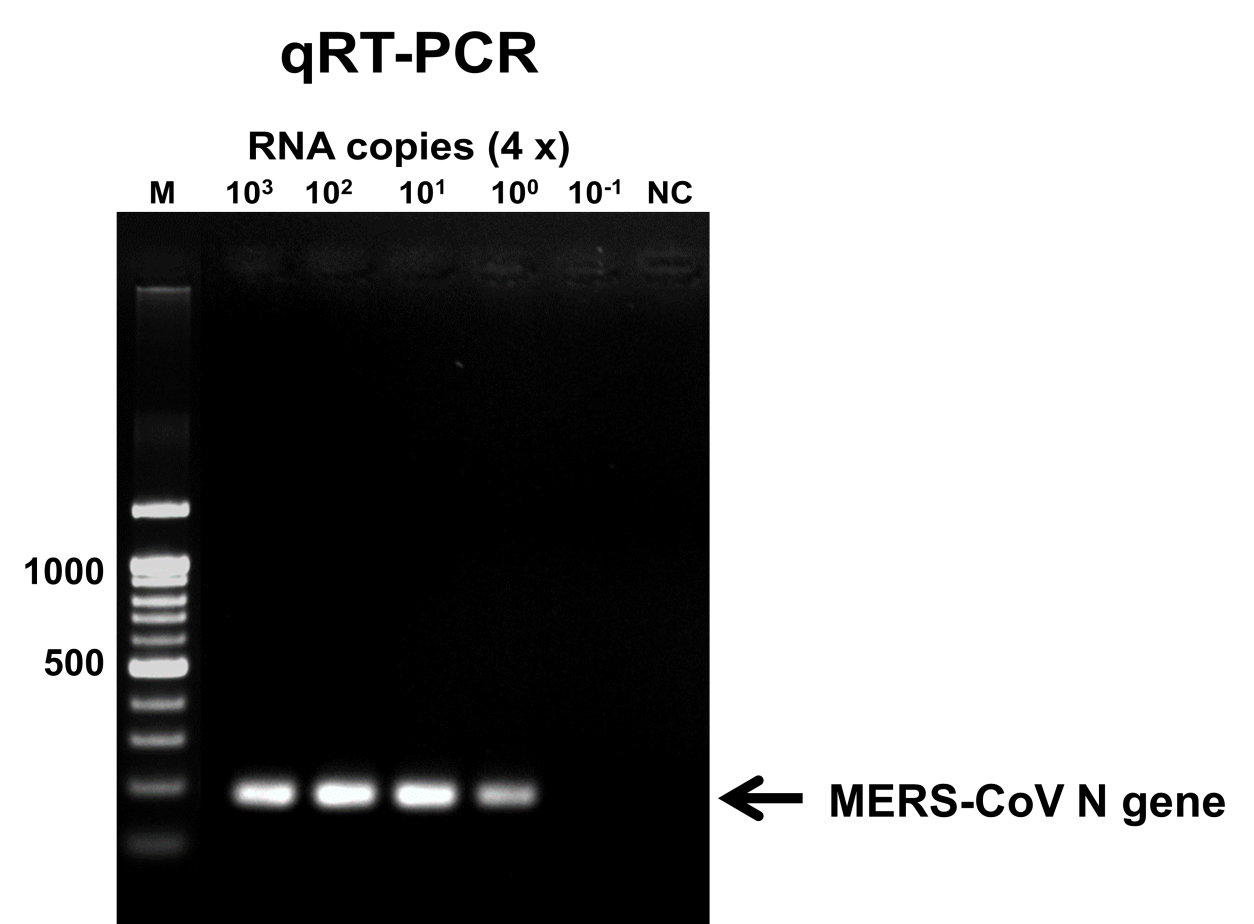


**Figure S4.** Real-time qRT-PCR agarose gel analysis. qRT-PCR was performed using equal amount of RNA which was applied for RT-LAMP reaction. MERS-CoV N gene was specifically amplified (arrow, 191 bp).

**.**


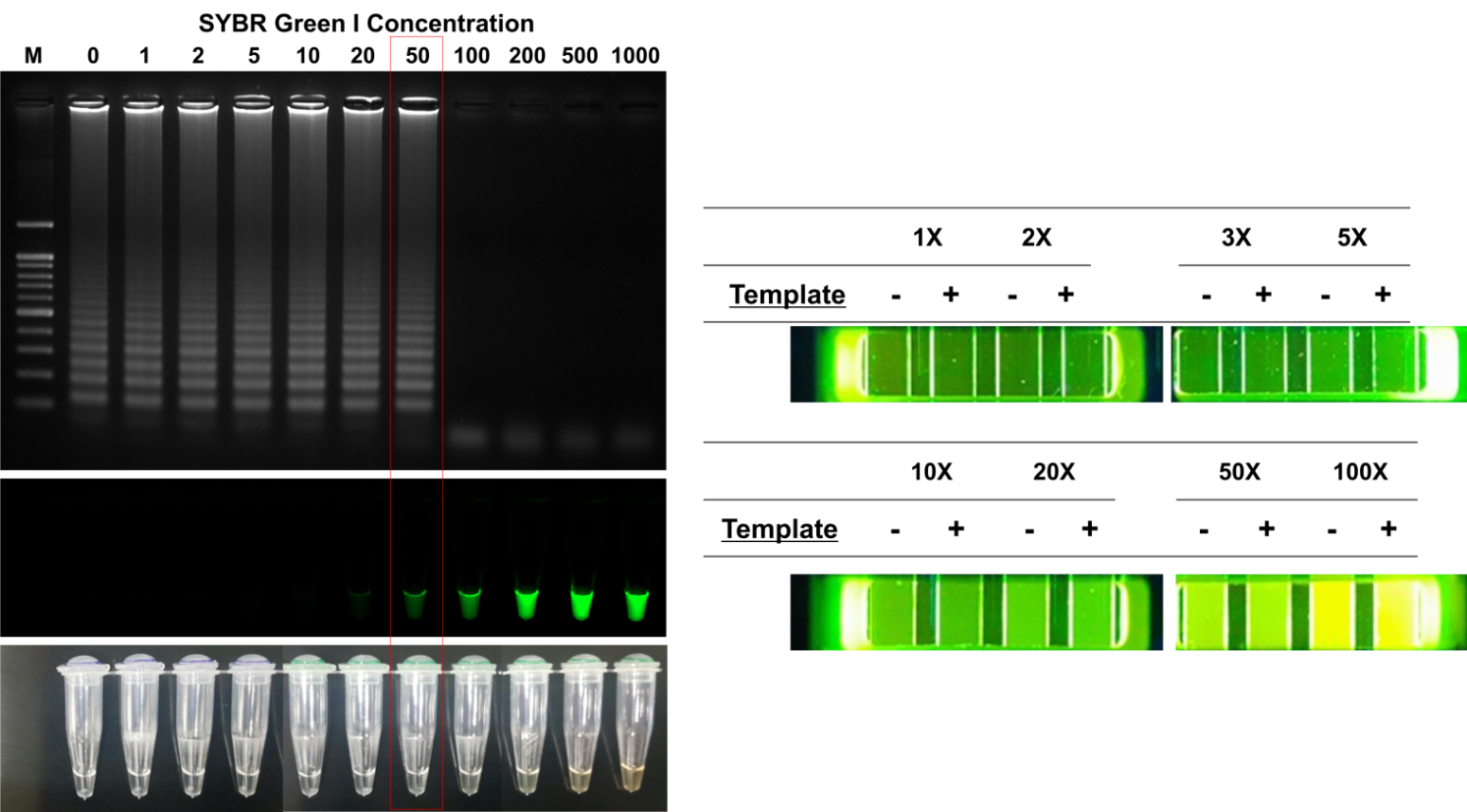


**Figure S5.** SYBR-green I activity for one-pot RT-LAMP. Although RT-LAMP amplification was successfully realized in a range from 0 ~ 50 X concentrated SYBR-green I, high concentrations (100 ~ 1000 X) inhibited the LAMP amplification. One-pot RT-LAMP experiments in a commercialized polymer-microchamber showed that there was no signal difference between positive and negative amplification (1, 2, 3, 5, 10, 20, 50, and 100X concentration) because of a high noise-background of SYBR-green I itself. This indicated that the one-pot RT-LAMP with SYBR Green-I failed to represent any significant correlation between positive and negative amplification response.


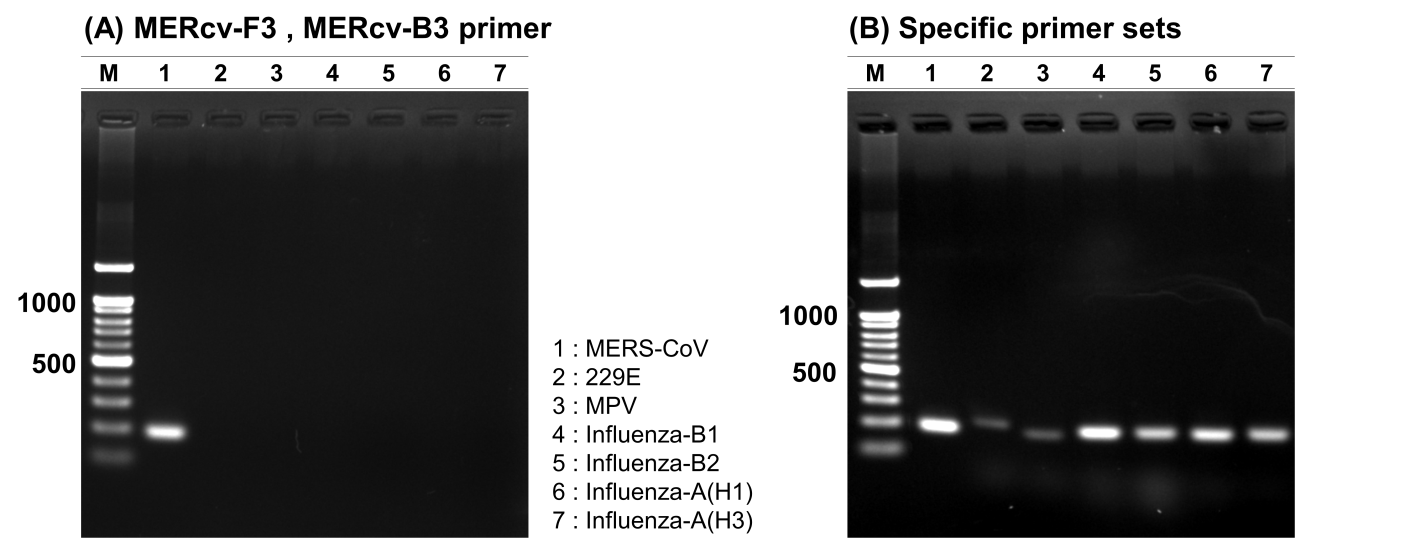


**Figure S6.** Verification of RNA samples and specific primers of the MERS-CoV and the other respiratory pathogens (A) RNA sample of MERS-CoV and the other respiratory pathogens was amplified by one-step RT-PCR using MERcv-F3 and MERcv-B3 primers designated for MERS-CoV detection (See Table 1). (B) RNA sample of MERS-CoV and the other respiratory pathogens was amplified by one-step RT-PCR using their specific primers (see Table S2). Each lane indicates a specific virus: lane 1, MERS-CoV Korean isolate; lane 2, Human coronavirus (HCoV)-229E; lane 3, Human metapneumovirus (HMPV); lane 4, B/Brisbane/60/2008 (Victoria lineage); lane 5, B/Phuket/3073/2013 (Yamagata lineage); lane 6, Influenza A virus (A/California/04/2009, H1N1); lane 7, influenza A virus (A/Perth/16/2009, H3N2). M, 100bp DNA ladder.

**Table S1.** LAMP primers for MERS-CoV

| **Candidate** | **Primer set** | **Sequence (5’ to 3’)** | **Position** | |
| --- | --- | --- | --- | --- |
| **Primer 1-T4**  **(P1-T4)** | **MERcv-F3** | GGAATGGAATTAAGCAACTGGC | 28828-28849 |  |
|  | **MERcv-B3** | CGCGAATTGTGTAACAATAGCT | 28997-29018 |  |
|  | **MERcv-FIP-T4** | ACAGCCCGGAATGGGAG**-(TTTT)-**GTGGTACTTCTACTACACTGGAA | 28896-28912+(T4 space)+28856-28878 |  |
|  | **MERcv-BIP-T4** | TAAGGATGGCATCGTTTGGGT**-(TTTT)-**TCATTGTTAGGGTTCCGCG | 28913-28933+(T4 space)+28975-28993 |  |
|  | **MERcv-LF** | TGCTGCTTCGGGTCCAG | 28879-28895 |  |
|  | **MERcv-LB** | GCGCCACTGATGCTCCTTC | 28945-28963 |  |
| **Primer 2**  **(P2)** | **MERcv-F3-2** | TGGAATTAAGCAACTGGCTCC | 28832-28852 |  |
|  | **MERcv-B3-2** | CGGGCGCGAATTGTGT | 29007-29022 |  |
|  | **MERcv-FIP-2** | AGCCCGGAATGGGAGTG-CAGGTGGTACTTCTACTACACT | 28894-28910+28853-28874 |  |
|  | **MERcv-BIP-2** | TTAAGGATGGCATCGTTTGGGT-TCATTGTTAGGGTTCCGCG | 28912-28933+28975-28993 |  |
|  | **MERcv-LF-2** | CTGCTTCGGGTCCAGTTCC | 28875-28893 |  |
|  | **MERcv-LB-2** | GCGCCACTGATGCTCCTTC | 28945-28963 |  |
| **Primer 2-T4**  **(P2-T4)** | **MERcv-F3-2** | TGGAATTAAGCAACTGGCTCC | 28832-28852 |  |
|  | **MERcv-B3-2** | CGGGCGCGAATTGTGT | 29007-29022 |  |
|  | **MERcv-FIP-1-T4** | AGCCCGGAATGGGAGTG**-(TTTT)-**CAGGTGGTACTTCTACTACACT | 28894-28910+(T4space)+28853-28874 |  |
|  | **MERcv-BIP-1-T4** | TTAAGGATGGCATCGTTTGGGT**-(TTTT)-**TCATTGTTAGGGTTCCGCG | 28912-28933+(T4space)+28975-28993 |  |
|  | **MERcv-LF-2** | CTGCTTCGGGTCCAGTTCC | 28875-28893 |  |
|  | **MERcv-LB-2** | GCGCCACTGATGCTCCTTC | 28945-28963 |  |

**Table S2.** Specific primers for detecting respiratory viruses

| **Virus** | **Primers** | **Sequence (5’ to 3’)** | **Position** | **Target viral gene** |
| --- | --- | --- | --- | --- |
| **229E** | **F** | GCAAAACTTACTACAGTTAC | 22586-22605 | S |
|  | **R** | GGAGGTACTCAACTTGAACT | 22787-22808 |  |
| **MPV** | **F** | GGRATAACACCAGCAATATC | 643-653 | M |
|  | **R** | GGCAGCTGSACCATGTAAAT | 802-821 |  |
| **Influenza B** | **F** | GGAGGACTACCACAAAGTG | 757-775 | HA |
|  | **R** | AATCCACCGTATTTTTCGTG | 934-953 |  |
| **Influenza A(H1)** | **F** | AGCAAGAAGTTCAAGCCG | 670-687 | HA |
|  | **R** | CGTGAACTGGTGTATCTGAA | 852-871 |  |
| **Influenza A(H3)** | **F** | ATAGTAAAACCGGGAGACAT | 754-773 | HA |
|  | **R** | GGCCCCATATGTGATCCT | 943-960 |  |
